# The Ethanol Extract of the Inner Bark of *Caesalpinia pyramidalis* (Tul.) Reduces Urinary Bladder Damage during Cyclophosphamide-Induced Cystitis in Rats

**DOI:** 10.1155/2013/694010

**Published:** 2013-11-20

**Authors:** Janaína P. Moraes, Denyson S. Pereira, Alexandre S. Matos, Danielle G. Santana, Cliomar A. Santos, Charles S. Estevam, Ricardo Fakhouri, Waldecy de Lucca Junior, Enilton A. Camargo

**Affiliations:** ^1^Department of Physiology, Federal University of Sergipe (UFS), 49100-000 São Cristóvão, SE, Brazil; ^2^Department of Medicine, Federal University of Sergipe (UFS), 49060-000 Aracaju, SE, Brazil; ^3^Department of Morphology, Federal University of Sergipe (UFS), 49100-000 São Cristóvão, SE, Brazil

## Abstract

Hemorrhagic cystitis (HC) is a common side effect of cyclophosphamide therapy, which deserves new therapeutic strategies, such as those based on natural products. The ethanol extract of the inner bark of *Caesalpinia pyramidalis* (Tul.) (EECp) possesses anti-inflammatory, antinociceptive, and antioxidant activities as previously showed by our group. We have investigated the effect of EECp on the cyclophosphamide-induced HC. Cystitis was induced in male Wistar rats by the injection of cyclophosphamide. These animals were pretreated with EECp (100–400 mg/kg), vehicle, or mesna. Myeloperoxidase activity and malondialdehyde formation were measured in urinary bladder and other tissues. Bladder edema and histopathological alterations and serum nitric oxide metabolites concentration NO_*x*_^−^ were also evaluated. Treatment with EECp (100–400 mg/kg) or mesna impaired the increase of myeloperoxidase activity in urinary bladder and the serum NO_*x*_^−^ induced by cyclophosphamide but did not reduce edema in this tissue, as did mesna. Total histological score was reduced by EECp (100 mg/kg). Lung myeloperoxidase activity, which was increased by cyclophosphamide, was decreased significantly by EECp (400 mg/kg). EECp also diminished the malondialdehyde formation in bladder, lung, and spleen, although these parameters were not affected by cyclophosphamide. These results indicate that EECp reduced urinary bladder damage during cyclophosphamide-induced HC in rats.

## 1. Introduction

The treatment of many neoplasic or nonneoplasic conditions with oxazophorines, mainly cyclophosphamide and ifosfamide, leads to hemorrhagic cystitis as an important side effect [1, 2]. Cyclophosphamide is an alkylating agent used, for example, to treat breast cancer, B-cell lymphoma, leukemia, rheumatoid arthritis, and systemic lupus erythematosis and in bone marrow transplantation [1, 3, 4]. This compound acts by cross-linking strands of DNA, thus preventing the division of cells, but its hepatic metabolism forms acrolein, between other metabolites, that is recognized for the ulceration, hemorrhage edema, and necrosis of the urothelium during its excretion by the urine [4, 5].

The treatment of cystitis is usually performed by the use of mesna (2-mercaptoethane sulfonate sodium), which can bind to and inactivate acrolein in the urinary bladder or other parts of urinary tract [2, 6]. Other options to treat hemorrhagic cystitis are desirable and studies in experimental animals have shown that nonsteroidal anti-inflammatory agents, corticosteroids, or nitric oxide synthase inhibitors may exert protective effects on the urinary tract in cyclophosphamide or ifosfamide-induced cystitis [7–10].

Another opportunity to develop new options to treat cystitis is the use of natural products. In this regard, many studies have shown that extracts of medicinal plants or compounds isolated from these plants can reduce cystitis induced by oxazophorines in rodents. For example, Boeira et al. [11] recently demonstrated that the hydroalcoholic extract of *Phyllanthus niruri* (L.) and the isolated compounds quercetin, rutin, and gallic acid were effective in reducing cyclophosphamide-induced hemorrhagic cystitis in mice. Also, the alcoholic extract of *Ipomoea obscura* (Linn.) was shown to ameliorate cyclophosphamide-induced bladder and renal toxicities [12] and the essential oil of *Satureja khuzestanica* (Jamzad) protected rats from cyclophosphamide-induced hemorrhagic cystitis, mainly due to antioxidant capacity [13]. 

*Caesalpinia pyramidalis* Tul. (Fabaceae) is an endemic tree of the Northeast region of Brazil that is popularly known as “catingueira.” Parts of this plant, especially the inner bark or leaves, are traditionally used because of their anti-inflammatory, diuretic, dyspeptic, digestive, antipyretic, and expectorant effects [14]. Santos et al. [15] have demonstrated that the ethanol extract of the inner bark of this plant possesses anti-inflammatory and antinociceptive activities in rodents, as well as antioxidant capacity. More recently, Santana et al. [16] have showed that this extract reduced pancreatic inflammation and oxidative stress and hyperamylasemia and abdominal hyperalgesia observed in rats with common bile duct obstruction-induced acute pancreatitis. Collectively, these studies suggest that *C. pyramidalis* has a potential for the treatment of inflammatory and painful conditions of clinical relevance, in which oxidative stress is an important feature. In this way, the present study was designed to investigate the potential of *C. pyramidalis* to reduce hemorrhagic cystitis in rats.

## 2. Material and Methods

### 2.1. Drugs and Reagents

Cyclophosphamide, mesna, hexadecyltrimethylammonium bromide, *o*-dianisidine hydrochloride, and Türk solutions were purchased from Sigma (USA). Isoflurane (Isoforine) was obtained from Cristália, Itapira, SP, Brazil. Other reagents were obtained from Merck.

### 2.2. Plant Material and Preparation of the Inner Bark Ethanol Extract

The inner bark of *Caesalpinia pyramidalis* was collected at the Xingó Village, Canindé de São Francisco, Sergipe State, Brazil (09°66′00′′ S, 37°78′94′′ W). A specimen was identified by the botanist Dr. Ana Paula Nascimento Prata, Department of Biology at the Federal University of Sergipe, and deposited in the Herbarium of this institution (São Cristóvão, Sergipe, Brazil) under the registration number ASE 13,164. The inner bark was dried at 40°C with forced air for 2 days and subsequently powdered (2,840 g) and extracted by maceration at room temperature with 90% ethanol for 5 days. The extract was filtered in vacuum, and the solvent was removed using a rotary evaporator (45°C). The percentage of EECp yield was 2.6% (73.8 g). A chromatographic analysis of EECp was previously performed by our group [16].

### 2.3. Animals

Male Wistar rats (220–270 g, *n* = 8/group) were obtained from the Animal Center of the Federal University of Sergipe. Animals were maintained at 21 ± 2°C with free access to food (Purina) and filtered water under a 12 : 12 h light/dark cycle. The animals were deprived of food for 8 h before the experiment, but had free access to water. All experimental procedures were conducted in accordance with the guidelines of the Brazilian College of Animal Experimentation and were approved by the Ethics Committee for Animal Use in Research at the Federal University of Sergipe (protocol number 055/09), which was conducted in accordance with the internationally accepted principles for laboratory animal use and care.

### 2.4. Induction of Hemorrhagic Cystitis

Hemorrhagic cystitis was induced by the injection of cyclophosphamide (200 mg/kg, 5 mL/kg, i.p.), according to previous studies [10, 17]. Control animals received saline (0.9%, 5 mL/kg, i.p.).

After 24 hours of cyclophosphamide injection, animals were anesthetized with inhalatory isoflurane (3%). Samples of blood were collected from the abdominal vein; animals were exsanguinated and submitted to transcardiac perfusion with saline 0.9% plus heparin (5 U/L). Urinary bladder tissue and samples of lung, spleen, liver, and kidney were collected for biochemical dosages.

### 2.5. Experimental Design

The following experimental groups were used: (i) Vehicle + Saline group: animals were orally pretreated with vehicle (tween 80, 5%, 10 mL/kg) 1 hour prior to injection of saline; (ii) Vehicle + cyclophosphamide group: animals were orally pre-treated with vehicle (tween 80, 5%, 10 mL/kg) 1 hour prior to injection of cyclophosphamide; (iii) EECp + cyclophosphamide group: animals were orally pre-treated with EECp (100, 200 or 400 mg/kg) 1 hour prior to injection of cyclophosphamide; (iv) Mesna + cyclophosphamide group: animals were treated with i.p. administration of mesna (40 mg/kg, 1 mL/kg), 5 min before and at 4 and 8 hours after injection of cyclophosphamide [7].

### 2.6. Determination of Urinary Bladder Edema

Edema of urinary bladder was estimated by weighting this tissue after dissection and expressing values as the result of urinary bladder weight (mg) divided by the whole body weight of animals (g) [18].

### 2.7. Determination of Biochemical and Inflammatory Parameters

For myeloperoxidase (MPO) activity determination, samples of urinary bladder, lung, spleen, liver, and kidney were collected, weighted, and homogenized with potassium phosphate buffer (50 mM, pH 6.0 containing 0.5% of hexadecyltrimethylammonium bromide), and 1 mL aliquots of the homogenates were incubated at 60°C for 2 h (for inactivation of catalase) and centrifuged (2 min, 8,000 ×g, 4°C). In a 96-well plate, aliquots of supernatant were incubated with a solution of *o*-dianisidine hydrochloride (0.167 mg/mL containing 0.005% H_2_O_2_). The MPO activity was measured kinetically in a microliter plate scanner (Labsystem Multiskan) at 460 nm and intervals of 15 s over a period of 5 min. Results were expressed as units of MPO per mg tissue (UMPO/mg tissue). An UMPO was considered as the amount of enzyme that degrades 1 mmol of hydrogen peroxide/min [18, 19].

For determination of thiobarbituric acid reactive substances (TBARS), samples of urinary bladder, lung, spleen, liver, and kidney were weighted and homogenized in potassium phosphate buffer (50 mM, pH 7.4) containing butylated hydroxytoluene (BHT, 12.6 mM). Then, aliquots of the homogenate (in duplicate) were incubated (90°C, 45 min) with thiobarbituric acid (TBA; 0.37%) in an acidic solution (trichloroacetic acid at 15% and hydrochloric acid at 0.25 N). At the end of incubation, the homogenates were centrifuged (5 min, 8,000 ×g), and aliquots from the supernatants were extracted with n-butanol, followed by stirring in a vortex for 30 s and further centrifugation (2 min, 8,000 ×g). The supernatant absorbance was measured at 535 nm in a microplate reader (corrected by the values of absorbance at 572 nm). The results were calculated using a molar extinction coefficient of 1.55 × 10^5^ M^−1^ cm^−1^ and expressed as pmol of malondialdehyde (MDA) formed per mg of tissue [16, 20].

Total nitrate/nitrite (NO_*x*_^−^) concentration was determined in serum submitted to ultrafiltration (10 kDa; Microcon centrifugal filter units) using the Griess reaction for nitrite, after the nitrate reductase-catalyzed reduction of nitrate to nitrite, as previously described [16].

Total leukocyte counts were performed in aliquots of 20 *μ*L of peripheral blood taken from the tail vein of anesthetized rats, immediately before euthanasia. The total leukocyte count was performed in a Neubauer chamber. Results were expressed as number of leukocytes/mL of peripheral blood [16].

### 2.8. Histopathological Analysis

After euthanasia, bladders were removed, fixed in formalin, embedded in paraffin, and cut into 5 *μ*m sections, which were stained with hematoxylin and eosin for morphological analysis (*n* = 5 animals). To evaluate the effect of EECp (100 or 400 mg/kg) on cyclophosphamide-induced cystitis, hemorrhage, edema, and leukocyte infiltration, mucosal ulceration and erosion were scored to reflect the severity of cystitis as follows: 0 = normal, 1 = mild changes, 2 = moderate changes, and 3 = severe changes. For each bladder, four sections were cut and scored.

### 2.9. Statistical Analysis

Results were expressed as mean ± SEM and analyzed by one-way analysis of variance (ANOVA) followed by Bonferroni's *post hoc* test by using GraphPad Prism software (version 4.0). *P* < 0.05 was considered significant. For histological analysis, the results were expressed as the median (min–max) and analysis was performed first by using the nonparametric Kruskal-Wallis test to discover whether there was any difference between groups. The Mann-Whitney *U* test was then performed to analyze two groups consecutively.

## 3. Results

### 3.1. Effect of EECp on Urinary Bladder Alterations during Cyclophosphamide-Induced Cystitis

**Figure 1:**
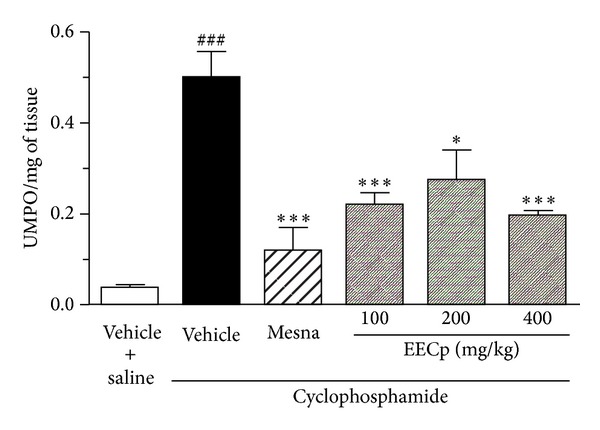
The ethanol extract of *Caesalpinia pyramidalis* (EECp) decreases the myeloperoxidase (MPO) activity in bladder of rats during cyclophosphamide-induced cystitis. Animals were treated with EECp (100–400 mg/kg, p.o.), vehicle, or mesna (40 mg/kg, i.p.) and injected with cyclophosphamide (200 mg/kg, i.p.). After 24 h, the MPO activity was determined in urinary bladder homogenate and was expressed as units (U) of MPO/mg of tissue. ^###^ *P* < 0.001 versus vehicle + saline; *P < 0.05 or ***P < 0.001 versus vehicle + cyclophosphamide.

Injection of cyclophosphamide (200 mg/kg) markedly augmented the MPO activity in urinary bladder tissue, when compared with saline group (*P* < 0.001). The treatment with EECp significantly reduced this activity (*P* < 0.001 for 100 or 400 mg/kg and *P* < 0.05 for 200 mg/kg, *n* = 8; Figure 1).

**Figure 2:**
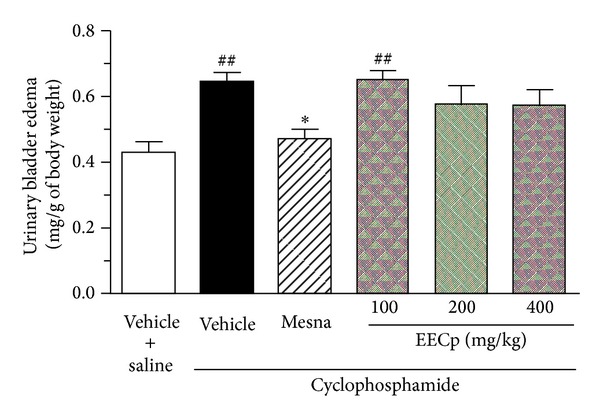
Urinary bladder weight of rats with cyclophosphamide-induced cystitis submitted to treatment with the ethanol extract of *Caesalpinia pyramidalis* (EECp). Animals were treated with EECp (100–400 mg/kg, p.o.), vehicle, or mesna (40 mg/kg, i.p.) and injected with cyclophosphamide (200 mg/kg, i.p.). After 24 h, the urinary bladder weight was measured and expressed as mg/g of whole body weight of animals. ^##^ *P* < 0.01 versus vehicle + saline; *P < 0.05 versus vehicle + cyclophosphamide.

Additionally, administration of cyclophosphamide to rats significantly increased the urinary bladder weight, when compared with saline-treated rats (*P* < 0.01), which was inhibited (*P* < 0.05) by the treatment with mesna (40 mg/kg). The oral administration of EECp (100–400 mg/kg) did not change the urinary bladder weight, when compared to vehicle-treated rats (Figure 2).

Although the injection of cyclophosphamide did not cause MDA formation (62 ± 17 pmol of MDA/mg of tissue, *n* = 8) when compared with saline group (93 ± 21 pmol of MDA/mg of tissue, *n* = 8), the EECp significantly decreased the MDA content in urinary bladder tissue homogenate (*P* < 0.05 for each dose) at the doses of 100, 200, or 400 mg/kg (27 ± 13, 48 ± 13 and 23 ± 12 pmol of MDA/mg of tissue, resp., *n* = 8) when compared with vehicle-treated animals.

### 3.2. Effect of EECp on MPO Activity and MDA Formation in Other Tissues, Total Peripheral Blood Leukocyte Counts and Serum NO_*x*_^−^ Levels

The MPO activity in the lung tissue of rats injected with cyclophosphamide was also increased in about 120% (*P* < 0.05), when compared with saline-injected group. The pretreatment with EECp decreased significantly (*P* < 0.05) this activity at doses of 200 or 400 mg/kg, which was not observed for 100 mg/kg, when compared with cyclophosphamide group. Treatment with mesna did not alter MPO activity in lung, although there was a tendency to lower values in this group (Table 1). In this tissue, there was no detectable difference in MDA formation after the cyclophosphamide injection, however, both the EECp (200 or 400 mg/kg) and mesna treatments significantly decreased the MDA formation in lung (*P* < 0.05, Table 1).

In the spleen tissue there was no differences in the MPO activity or MDA formation after 24 h of the administration of cyclophosphamide to rats. However, it was also observed that EECp significantly reduced the MDA formation in spleen at the dose of 400 mg/kg, without affecting MPO activity in this tissue. Mesna did not modify these parameters in spleen (Table 1).

Liver and kidney tissue MPO activity and MDA formation were also measured and very low MPO activity was found in these tissues (lower than 0.05 UMPO/mg of tissue), with no difference between the groups evaluated (data not shown).

Total peripheral blood leukocyte counts were decreased significantly by the cyclophosphamide administration (6.7 ± 0.9 cells/mL), when compared with saline group (13.2 ± 1.0 cells/mL; *P* < 0.001). This reduction was not significantly affected by the treatment with mesna (5.8 ± 1.6 cells/mL) or EECp at 100, 200, or 400 mg/kg (4.6 ± 0.9, 4.8 ± 0.5, and 5.3 ± 1.0 cells/mL, resp.).

Serum concentrations of NO_*x*_^−^ were also augmented by the injection of cyclophosphamide (59.8 ± 4.9 *μ*M), when compared to saline-injected group (10.3 ± 2.2 *μ*M; *P* < 0.001). Treatment with EECp significantly reduced these concentrations at the doses of 100 mg/kg (33.1 ± 8.8 *μ*M, *P* < 0.05) or 400 mg/kg (24.4 ± 5.7 *μ*M, *P* < 0.01), as did the treatment with mesna (20.8 ± 5.2 *μ*M; *P* < 0.01). The treatment with the dose of 200 mg/kg was not tested for this parameter.

### 3.3. Effect of EECp on the Histopathological Alterations Induced by Cyclophosphamide

**Figure 3:**
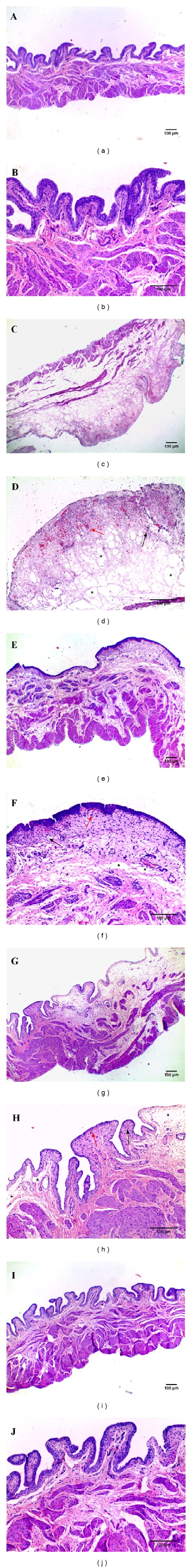
Light microscopy of the bladders of rats with cystitis. Original magnification of 10-fold (Panels (a), (c), (e), (g), and (i)) or 40-(Panels (b), (d), (f), (h), and (j)); hematoxylin and eosin staining. Panels (a) and (b) show control saline + vehicle rats with normal tissue histology. Panels (c) and (d) demonstrate that injection of cyclophosphamide induced mucosal erosion and ulceration, severe edema in the submucosal region (∗), hemorrhagic foci (red arrows), and leukocyte infiltration (black arrows). Panels (e) and (f) indicate that EECp, at 100 mg/kg, reduced the cyclophosphamide-induced morphological alterations, affecting mainly the leukocyte infiltration and mucosal erosion/ulceration, which was not so clearly observed in the urinary bladders of EECp (400 mg/kg)-pretreated rats (Panels (g) and (h)) that still presented mucosal erosion, ulceration, edema in the submucosal region, and hemorrhagic foci. A slight leukocyte infiltration reduction was observed in these animals ((g) and (h)) when compared with animals pretreated with vehicle and injected with cyclophosphamide ((c) and (d)). Panels (i) and (j) show that mesna pretreatment almost completely reverted the damage induced by cyclophosphamide.

The administration of cyclophosphamide to rats caused histopathological alterations in the urinary bladder, which were characterized by moderate-severe edema of lamina propria and hemorrhage and severe mucosal ulceration and erosion, as well as leukocyte infiltration (Table 2 and Figures 3(c) and 3(d)), when compared with the saline-injected animals that presented normal pattern (Figures 3(a) and 3(b)). Presence of leukocyte in cyclophosphamide-injected and vehicle-treated animals was primarily characterized by polymorphonuclear cells in the lamina propria.

The treatment with EECp at 100 mg/kg decreased the leukocyte infiltration in the urinary bladder in a significant manner (*P* < 0.05, Table 2), but only showed a tendency to reduce the other parameters analyzed, although it diminished the total score of morphological alterations induced by cyclophosphamide (Table 2 and Figures 3(e) and 3(f)). The dose of 400 mg/kg also tended to improve the histology of urinary bladder, but it was not as effective as the lower dose of EECp used (Table 2 and Figures 3(g) and 3(h)). The dose of 200 mg/kg was not performed for this analysis. Treatment with mesna significantly reduced the effect of cyclophosphamide for all parameters evaluated (Table 2 and Figures 3(i) and 3(j)).

## 4. Discussion

Treatment with cyclophosphamide, an alkylating agent, is largely known to cause urotoxic effects [1, 2]. In this study, cyclophosphamide injection to rats caused edema, increase of MPO activity, and histopathological alterations in the urinary bladder tissue, as expected [21], which were partially reduced by treatment with EECp, with the exception of bladder edema. 

The pharmacological activity of *Caesalpinia pyramidalis* was described recently by a previous study [15], which demonstrated that treatment with EECp, at the same doses used in the present study, decreased inflammation in carrageenan-induced rat paw edema or mice peritonitis and inhibited the nociception induced by various stimuli and possessed in vitro antioxidant properties. As these properties would have value to treat hemorrhagic cystitis, we tested here the effects of this extract. In this way, although the pretreatment with EECp did not alter the urinary bladder edema, it reduced the neutrophil accumulation in this tissue, as measured by the MPO activity. This finding was confirmed for the dose of 100 mg/kg of EECp, through the histological analysis. Besides the inhibition of leukocyte infiltration, this dose of EECp tended to reduce the edema of the lamina propria, hemorrhagia, and the mucosal erosion and ulceration of urinary bladder. Interestingly, the higher dose utilized of EECp did not show a significant improvement of urinary bladder tissue histology, although a tendency for lower scores was observed. Taken together, these results suggest that even lower doses of EECp could be used in rats. 

Interestingly, we did not detect lipid peroxidation in the urinary bladders, 24 h after the injection of cyclophosphamide, which contrasts with the findings by others [22–24], but it does not exclude the possibility that peroxidation may have occurred at previous experimental time points, which were not evaluated. In spite of this finding, we observed a reduced MDA formation in the bladders from animals treated with EECp, which enable us to speculate that the inhibitory effect of neutrophil infiltration is related to the diminishment of MDA formation by EECp. As a matter of interest, a similar link between reduced MDA formation and MPO activity was described by Santana et al. [16] in the model of acute pancreatitis induced by the common bile duct obstruction in rats. These results further agree with the previous data from Santos et al. [15], showing in vitro antioxidant activity and anti-inflammatory effect of EECp. All together, these data indicate that EECp induced anti-inflammatory effect in urinary bladder during the cyclophosphamide-induced hemorrhagic cystitis model in rats.

An additional important finding of this study is that the EECp treatment is able to reduce the serum concentrations of metabolites of NO in rats with cystitis. Overproduction of NO is a characteristic of the inflammatory process and it was showed that inhibition of inducible nitric oxide synthase isoform by aminoguanidine has beneficial effects in the cyclophosphamide-induced cystitis in rodents [10]. The fact that EECp reduced the serum concentrations of NO metabolites is in accordance with the protective effects of this extract in the urinary bladder, especially regarding to the decrease of the MDA levels in this tissue. Also, it is plausible that reduced NO metabolite formation, induced by EECp, in other organs may contribute to this effect.

In this study, the effect of EECp was also evaluated in some tissues of rats injected with cyclophosphamide, like lung, spleen, liver, and kidney, or in the leukocyte count in peripheral blood. It is interesting to note that cyclophosphamide treatment increased the MPO activity in lung. This result is in accordance with previous studies that showed the potential of cyclophosphamide to cause lung toxicity [25, 26]. However, no lipid peroxidation was detected in lung tissue in our study, which contrasts with other authors' findings [27]. The EECp pretreatment decreased MPO activity in lungs, reinforcing its anti-inflammatory activity, and decreased basal values of MDA formation in this tissue. Accordingly, a study from our group showed that the lung neutrophil infiltration and lipid peroxidation secondary to acute pancreatitis induced by bile duct ligation in rats was also reduced by treatment with EECp [16]. Spleen, liver and kidney MPO activities were not affected by cyclophosphamide or EECp, but EECp decreased the MDA formation in rat spleen, without affecting liver, and kidney lipid peroxidation. This may be linked with the antioxidant activity presented by EECp [15, 16], but it can be argued that the actual importance of this finding is related to the fact that administration of EECp, in the dose range used, does not lead to the injury of these tissues, especially liver and kidney. By comparison, previous data from our group showed that the treatment with the ethanol extract of *Sideroxylon obstusifolium*, in the same way that was used in the present study, increased lipid peroxidation in spleen and urinary bladder of rats [18].

Cyclophosphamide is also well known by its damaging effects in the hematopoietic stem cells and precursor cells and to the hematopoietic microenvironment, leading to myelosuppression [28, 29]. In our study, we detected a decrease in the total leukocyte number in peripheral blood that was not changed by EECp, indicating that this extract does not have any effect on the negative hematopoietic effects of cyclophosphamide.

The components of EECp are still poorly characterized, but the study by Santana et al. [16] has strongly suggested that rutin, a well-known flavonoid, is present in this extract. As a matter of interest, rutin, present in the hydroalcoholic extract of *Phyllanthus niruri* (Euphorbiaceae), was shown to diminish the urinary bladder inflammation during cystitis induced by cyclophosphamide in mice [11]. Whether rutin is presented in EECp in a concentration high enough to reach biological significance for the effects of this extract is still not established, and the possibility that other compounds would contribute to these protective actions cannot be discharged.

In summary, we provided here evidence that demonstrate that EECp decreases cyclophosphamide-induced pathological alterations of urinary bladder, as neutrophil infiltration. This extract also does not have any deleterious effects, detected in terms of MPO activity and MDA formation, in lung, spleen, kidney, and liver. This indicates that EECp has a potential to treat cystitis induced by oxazophorines.

## Figures and Tables

**Table 1:** Effect of the ethanol extract of *Caesalpinia pyramidalis* (EECp) on the myeloperoxidase (MPO) activity and lipid peroxidation of lung and spleen of rats during cyclophosphamide (CP)-induced cystitis.

Tissue	Group	MPO (UMPO/mg of tissue)	MDA (pmol of MDA/mg of tissue)
Lung	Vehicle + Saline	6.6 ± 1.7	35.0 ± 8.5
	Vehicle + CP	14.6 ± 1.7^#^	40.2 ± 5.5
	Mesna + CP	7.7 ± 1.6	13.1 ± 4.8*
	EECp (100 mg/kg) + CP	7.9 ± 2.5	52.8 ± 12.3
	EECp (200 mg/kg) + CP	6.4 ± 1.4*	17.3 ± 4.3*
	EECp (400 mg/kg) + CP	6.3 ± 1.2*	12.7 ± 5.6*

Spleen	Vehicle + Saline	16.3 ± 2.0	35.1 ± 7.2
	Vehicle + CP	20.7 ± 1.1	43.1 ± 10.7
	Mesna + CP	15.3 ± 1.7	18.9 ± 6.7
	EECp (100 mg/kg) + CP	17.2 ± 3.0	35.2 ± 16.1
	EECp (200 mg/kg) + CP	20.9 ± 2.7	22.8 ± 11.3
	EECp (400 mg/kg) + CP	27.1 ± 2.4	2.7 ± 2.0*

Rats were treated with EECp (100–400 mg/kg), mesna (40 mg/kg), or vehicle and injected with saline or CP (200 mg/kg). After 24 h of induction, the MPO activity and malondialdehyde (MDA) formation were determined in the lung or spleen tissues. Data are expressed as mean ± SEM for *n* = 8 rats. ^#^ *P* < 0.05 versus respective vehicle + saline group; *P < 0.05 versus respective vehicle + CP group.

**Table 2:** Effect of ethanol extract of *Caesalpinia pyramidalis* (EECp) on the histopathological parameters during cyclophosphamide (CP)-induced cystitis in rats.

Group	Edema	Hemorrhage	Erosion and ulceration	Leukocyte	Total score
Saline + Vehicle	0 (0-0)	0 (0-0)	0 (0-0)	0 (0-0)	0 (0-0)
CP + Vehicle	2 (1–3)^#^	2 (1–3)^#^	3 (2-3)^#^	3 (3-3)^#^	11 (9–12)^#^
CP + Mesna	0 (0-1)*	0 (0-1)*	1 (1-2)*	0 (0-0)*	2 (2–4)*
CP + EECp (100 mg/kg)	1 (0–2)	1 (0–2)	2 (2-3)	1 (1-2)*	6 (4–8)*
CP + EECp (400 mg/kg)	1 (0–2)	1 (0–2)	2 (1–3)	3 (1–3)	9 (3–11)

Data are shown as median (min–max) for *n* = 5 animals. ^#^ *P* < 0.05 versus respective saline + vehicle group and *P < 0.05 versus respective CP + vehicle group.

## References

[1] Ratliff TR, Williams RD (1998). Hemorrhagic cystitis, chemotherapy, and bladder toxicity. *The Journal of Urology*.

[2] Korkmaz A, Topal T, Oter S (2007). Pathophysiological aspects of cyclophosphamide and ifosfamide induced hemorrhagic cystitis; implication of reactive oxygen and nitrogen species as well as PARP activation. *Cell Biology and Toxicology*.

[3] Perini P, Calabrese M, Rinaldi L, Gallo P (2008). Cyclophosphamide-based combination therapies for autoimmunity. *Neurological Sciences*.

[4] Mukhtar S, Woodhouse C (2010). The management of cyclophosphamide-induced haematuria. *BJU International*.

[5] Zhang J, Tian Q, Sui YC (2005). Metabolism and transport of oxazaphosphorines and the clinical implications. *Drug Metabolism Reviews*.

[6] Kehrer JP, Biswal SS (2000). The molecular effects of acrolein. *Toxicological Sciences*.

[7] Morais MM, Belarmino-Filho JN, Brito GAC, Ribeiro RA (1999). Pharmacological and histopathological study of cyclophosphamide-induced hemorrhagic cystitis—comparison of the effects of dexamethasone and Mesna. *Brazilian Journal of Medical and Biological Research*.

[8] Macedo FYB, Baltazar F, Almeida PRC (2008). Cyclooxygenase-2 expression on ifosfamide-induced hemorrhagic cystitis in rats. *Journal of Cancer Research and Clinical Oncology*.

[9] Abraham P, Rabi S (2009). Protein nitration, PARP activation and NAD+ depletion may play a critical role in the pathogenesis of cyclophosphamide-induced hemorrhagic cystitis in the rat. *Cancer Chemotherapy and Pharmacology*.

[10] Abraham P, Rabi S, Kulothungan P (2009). Aminoguanidine, selective nitric oxide synthase inhibitor, ameliorates cyclophosphamide-induced hemorrhagic cystitis by inhibiting protein nitration and PARS activation. *Urology*.

[11] Boeira VT, Leite CE, Santos AA (2011). Effects of the hydroalcoholic extract of Phyllanthus niruri and its isolated compounds on cyclophosphamide-induced hemorrhagic cystitis in mouse. *Naunyn-Schmiedeberg’s Archives of Pharmacology*.

[12] Hamsa TP, Kuttan G (2011). Protective role of Ipomoea obscura (L.) on cyclophosphamide-induced uro- and nephrotoxicities by modulating antioxidant status and pro-inflammatory cytokine levels. *Inflammopharmacology*.

[13] Rezvanfar MA, Farshid AA, Sadrkhanlou RA (2010). Benefit of *Satureja khuzestanica* in subchronically rat model of cyclophosphamide-induced hemorrhagic cystitis. *Experimental and Toxicologic Pathology*.

[14] De Fátima Agra M, De Freitas PF, Barbosa-Filho JM (2007). Synopsis of the plants known as medicinal and poisonous in Northeast of Brazil. *Brazilian Journal of Pharmacognosy*.

[15] Santos CA, Passos AMPR, Andrade FC (2011). Antinociceptive and anti-inflammatory effects of *Caesalpinia pyramidalis* in Rodents. *Brazilian Journal of Pharmacognosy*.

[16] Santana DG, Santos CA, Santos AD (2012). Beneficial effects of the ethanol extract of *Caesalpinia pyramidalis* on the inflammatory response and abdominal hyperalgesia in rats with acute pancreatitis. *Journal of Ethnopharmacology*.

[17] Kiuchi H, Takao T, Yamamoto K (2009). Sesquiterpene lactone parthenolide ameliorates bladder inflammation and bladder overactivity in cyclophosphamide induced rat cystitis model by inhibiting nuclear factor-kappaB phosphorylation. *Journal of Urology*.

[18] Pereira DS, Morais JP, Santana DG (2013). Effects of the ethanol extract of the inner bark of *Syderoxylum obtusifolium* in the cyclophosphamide-induced cystitis in rats. *Journal of Medicinal Plants Research*.

[19] Bradley PP, Priebat DA, Christensen RD, Rothstein G (1982). Measurement of cutaneous inflammation: estimation of neutrophil content with an enzyme marker. *Journal of Investigative Dermatology*.

[20] Bose R, Sutherland GR, Pinsky C (1989). Biological and methodological implications of prostaglandin involvement in mouse brain lipid peroxidation measurements. *Neurochemical Research*.

[21] Kanat O, Kurt E, Yalcinkaya U, Evrensel T, Manavoglu O (2006). Comparison of uroprotective efficacy of mesna and amifostine in cyclophosphamide- induced hemorrhagic cystitis in rats. *Indian Journal of Cancer*.

[22] Bhatia K, Ahmad F, Rashid H, Raisuddin S (2008). Protective effect of S-allylcysteine against cyclophosphamide-induced bladder hemorrhagic cystitis in mice. *Food and Chemical Toxicology*.

[23] Al-Yahya AA, Al-Majed AA, Gado AM (2009). Acacia senegal gum exudate offers protection against cyclophosphamide- induced urinary bladder cytotoxicity. *Oxidative Medicine and Cellular Longevity*.

[24] Abraham P, Isaac B, Ramamoorthy H, Natarajan K (2011). Oral glutamine attenuates cyclophosphamide-induced oxidative stress in the bladder but does not prevent hemorrhagic cystitis in rats. *Journal of Medical Toxicology*.

[25] Patel JM (1990). Metabolism and pulmonary toxicity of cyclophosphamide. *Pharmacology and Therapeutics*.

[26] Limper AH (2004). Chemotherapy-induced lung disease. *Clinics in Chest Medicine*.

[27] Sulkowska M, Sulkowski S, Skrzydlewska E, Farbiszewski R (1998). Cyclophosphamide-induced generation of reactive oxygen species. Comparison with morphological changes in type ii alveolar epithelial cells and lung capillaries. *Experimental and Toxicologic Pathology*.

[28] DeWys WD, Goldin A, Mantel N (1970). Hematopoietic recovery after large doses of cyclophosphamide: correlation of proliferative state with sensitivity. *Cancer Research*.

[29] Kusano K, Ebara S, Tachibana K (2004). A potential therapeutic role for small nonpeptidyl compounds that mimic human granulocyte colony-stimulating factor. *Blood*.

